# Supplementary material for: Relation of Dietary n-3 and n-6 Fatty Acid Intakes to Metabolic Syndrome in Middle-Aged People Depending on the Level of HbA1c: A Review of National Health and Nutrition Survey Data from 2014 to 2016
Source: Medicina (Kaunas). 2022 Jul 29;58(8):1017. doi: 10.3390/medicina58081017 (PMC9413490; doi:10.3390/medicina58081017)
Supplement: Supplementary file 1 [file medicina-58-01017-s001.zip › medicina-1776748-supplementary.pdf]

Table S1. Multivariate-adjusted odds ratios and 95% confidence intervals for the interactions between MetS and HbA1c groups (normal, high) according to n-3 and n-6 fatty acid intakes and gender.

	Odds Ratio——HbA1c <6.5 Tertiles of PUFA intake			Odds Ratio——HbA1c ≥6.5 Tertiles of PUFA intake		
**Men**	**1**	**2**	**3**	**1**	**2**	**3**
Participants (n)		1656			219	
**n-6 Fatty Acid**						
Range of intake(g/day)	<6.43	6.43—10.14	>10.14	<6.43	6.43—10.14	>10.14
Crude	1	0.50 (0.42—0.60)	0.55 (0.47—0.66)	1	2.19 (1.31—3.67)	2.37 (1.39—4.05)
Model 1	1	0.82 (0.64—1.04)	0.88 (0.70—1.12)	1	0.65 (0.32—1.32)	0.73 (0.36—1.49)
Model 2	1	0.94 (0.73—1.21)	1.08 (0.83—1.40)	1	0.61 (0.30—1.27)	0.64 (0.30—1.40)
Model 3	1	0.87 (0.65—1.16)	0.88 (0.57—1.36)	1	0.59 (0.23—1.50)	0.70 (0.15—3.17)
**n-3 Fatty Acid**						
Range of intake(g/day)	<0.87	0.87—1.40	>1.40	<0.87	0.87—1.40	>1.40
Crude	1	0.45 (0.38—0.54)	0.58 (0.49—0.69)	1	1.80 (1.10—2.94)	2.77 (1.59—4.82)
Model 1	1	0.70 (0.55—0.89)	0.89 (0.71—1.12)	1	0.47 (0.23—0.97)	0.76 (0.36—1.60)
Model 2	1	0.83 (0.64—1.07)	1.06 (0.82—1.36)	1	0.45 (0.22—0.94)	0.70 (0.32—1.56)
Model 3	1	0.74 (0.56—0.99)	0.81 (0.54—1.21)	1	0.41 (0.17—0.99)	0.77 (0.20—3.00)
**n-6/n-3 Ratio**						
Range of intake	<6.89	6.89—7.80	>7.80	<6.89	6.89—7.80	>7.80
Crude	1	0.48 (0.40—0.57)	0.55 (0.47—0.66)	1	2.48 (1.49—4.11)	3.92 (2.13—7.21)
Model 1	1	0.73 (0.58—0.93)	0.86 (0.68—1.08)	1	1.15 (0.59—2.25)	1.83 (0.87—3.86)
Model 2	1	0.84 (0.65—1.08)	0.91 (0.71—1.16)	1	1.23 (0.62—2.44)	1.93 (0.90—4.13)
Model 3	1	0.89 (0.68—1.16)	0.99 (0.73—1.33)	1	2.08 (0.92—4.71)	4.96 (1.73 -14.26)
**Women**	**1**	**2**	**3**	**1**	**2**	**3**
Participants (n)		2754			223	
**n-6 Fatty Acid**						
Range of intake(g/day)	<5.62	5.62—8.93	>8.93	<5.62	5.62—8.93	>8.93
Crude	1	0.31 (0.26—0.36)	0.23 (0.19—0.27)	1	3.70 (2.00—6.82)	3.93 (2.19—7.06)
Model 1	1	0.56 (0.46—0.68)	0.40 (0.33—0.49)	1	0.73 (0.32—1.63)	0.76 (0.35—1.69)
Model 2	1	0.77 (0.62—0.95)	0.64 (0.51—0.80)	1	0.92 (0.38—2.23)	1.07 (0.43—2.67)
Model 3	1	0.84 (0.66—1.08)	0.78 (0.52—1.15)	1	1.12 (0.38—3.28)	1.46 (0.28—7.62)

**n-3 Fatty Acid**						
Range of intake(g/day)	<0.8	0.80—1.29	>1.29	<0.8	0.80—1.29	>1.29
Crude	1	0.30 (0.26—0.35)	0.24 (0.21—0.29)	1	4.17 (2.22—7.82)	4.00 (2.23—7.19)
Model 1	1	0.55 (0.45—0.67)	0.44 (0.36—0.55)	1	0.87 (0.38—1.98)	0.82 (0.37—1.82)
Model 2	1	0.77 (0.62—0.95)	0.69 (0.55—0.86)	1	1.11 (0.46—2.70)	1.08 (0.45—2.59)
Model 3	1	0.85 (0.67—1.09)	0.84 (0.59—1.21)	1	1.32 (0.45—3.86)	1.28 (0.30—5.49)
**n-6/n-3 Ratio**						
Range of intake	<6.56	6.56—7.5	>7.50	<6.56	6.56—7.5	>7.50
Crude	1	0.27 (0.23—0.31)	0.32 (0.28—0.37)	1	3.12 (1.81—5.38)	3.86 (2.14—6.94)
Model 1	1	0.55 (0.45—0.68)	0.65 (0.53—0.79)	1	0.48 (0.21—1.09)	0.65 (0.29—1.48)
Model 2	1	0.75 (0.60—0.93)	0.90 (0.73—1.11)	1	0.65 (0.27—1.57)	0.99 (0.40—2.47)
Model 3	1	0.79 (0.63—0.99)	1.03 (0.80—1.32)	1	0.66 (0.27—1.65)	1.09 (0.39—3.09)

Adjusted for:
Model 1: age
Model 2: Model 1 + smoking status, alcohol use frequency, education level, employment status, lowest income quartile, and physical activity
Model 3: Model 2 + Daily intakes: energy, carbohydrates, protein, saturated fatty acids, monounsaturated fatty acids, cholesterol, and dietary fiber
